# Identification and Characterization of the *EXO70* Gene Family in Polyploid Wheat and Related Species

**DOI:** 10.3390/ijms20010060

**Published:** 2018-12-24

**Authors:** Jia Zhao, Xu Zhang, Wentao Wan, Heng Zhang, Jia Liu, Mengli Li, Haiyan Wang, Jin Xiao, Xiue Wang

**Affiliations:** State Key Laboratory of Crop Genetics and Germplasm Enhancement, Cytogenetics Institute, Nanjing Agricultural University/JCIC-MCP, Nanjing 210095, China; 2015201054@njau.edu.cn (J.Z.); 2018201061@njau.edu.cn (X.Z.); 2016201003@njau.edu.cn (W.W.); 2016201031@njau.edu.cn (H.Z.); 2018201063@njau.edu.cn (J.L.); 2016101134@njau.edu.cn (M.L.); hywang@njau.edu.cn (H.W.)

**Keywords:** EXO70, *Haynaldia villosa*, gene family, phylogenetic relationship, subcellular localization, expression profiling

## Abstract

The *EXO70* gene family is involved in different biological processes in plants, ranging from plant polar growth to plant immunity. To date, analysis of the *EXO70* gene family has been limited in *Triticeae* species, e.g., hexaploidy *Triticum aestivum* and its ancestral/related species. By in silico analysis of multiple *Triticeae* sequence databases, a total of 200 *EXO70* members were identified. By homologue cloning approaches, 15 full-length cDNA of *EXO70s* were cloned from diploid *Haynaldia villosa*. Phylogenetic relationship analysis of 215 *EXO70* members classified them into three groups (*EXO70.1*, *EXO70.2*, and *EXO70.3*) and nine subgroups (*EXO70A* to *EXO70I*). The distribution of most *EXO70* genes among different species/sub-genomes were collinear, implying their orthologous relationship. The *EXO70A* subgroup has the most introns (at least five introns), while the remaining seven subgroups have only one intron on average. The expression profiling of *EXO70* genes from wheat revealed that 40 wheat *EXO70* genes were expressed in at least one tissue (leaf, stem, or root), of which 25 wheat *EXO70* genes were in response to at least one biotic stress (stripe rust or powdery mildew) or abiotic stress (drought or heat). Subcellular localization analysis showed that ten EXO70-V proteins had distinct plasma membrane localization, EXO70I1-V showed a distinctive spotted pattern on the membrane. The 15 *EXO70-V* genes were differentially expressed in three tissue. Apart from *EXO70D2-V*, the remaining *EXO70-V* genes were in response to at least one stress (flg22, chitin, powdery mildew, drought, NaCl, heat, or cold) or phytohormones (salicylic acid, methyl jasmonate, ethephon, or abscisic acid) and hydrogen peroxide treatments. This research provides a genome-wide glimpse of the *Triticeae EXO70* gene family and those up- or downregulated genes require further validation of their biological roles in response to biotic/abiotic stresses.

## 1. Introduction

The exocyst complex is an evolutionarily conserved octameric tethering factor, which mediates the fusion of post-Golgi secretory vesicle with the plasma membrane (PM) and plays a major role in exocytosis [1,2]. EXO70 is a key member of the exocyst complex and has been found to be widely present in yeast, mammals and plants [3]. In yeast and mammals, the *EXO70* only has a single copy, while plants have multiple copies of *EXO70* genes [4] ranging from 21 to 47 *EXO70* members in potatoes (*Symphytum tuberosum*), *Arabidopsis*, *Populus trichocarpa* and rice [1,5,6]. The *EXO70s* of land plants possibly originated from three ancient *EXO70* genes and thus can be divided into three groups *EXO70.1, EXO70.2* and *EXO70.3*. They have been further duplicated independently in the moss, lycophyte and angiosperm lineages, and in the subsequent lineage-specific multiplications which are represented by nine subgroups (*EXO70A-EXO70I*) [4,7,8,9]. 

The function of *EXO70* has been extensively studied in yeast, and mammals [3,10,11,12,13,14,15]. In plants, *EXO70s* have been proven to play diverse roles in regulating plant growth and coping with adverse biotic/abiotic stresses. In *Arabidopsis*, *EXO70A1* has been implicated in a wide range of developmental processes, including the differentiation of tracheary elements [16,17], the development of seed coat, root hair and stigmatic papillae [18], the recycling of the auxin efflux carrier proteins (PIN1 and PIN2) [19] and the formation of the Casparian strip [20]. *EXO70C1* and *EXO70C2* regulated the polarized growth and maturation of the pollen tube [21,22]. *EXO70H4* regulates trichome cell wall maturation by mediating the secretion and accumulation of callose and silica [23,24]. The rice *OsEXO70A1* is necessary for vascular bundle differentiation and assimilation of mineral nutrients [5]. The legumes EXO70J7, EXO70J8 and EXO70J9 are members of an atypical subgroup of EXO70 proteins (EXO70J) that regulate leaf senescence and nodule formation [25]. In *Nicotiana benthamiana*, silencing all the paralogue genes in subgroups EXO70A (six), C (three), D (four) and G (six) resulted in a smaller leaf phenotype [6].

Evidence has accumulated for the critical role of *EXO70s* in plant-pathogen interactions or responses to abiotic stresses. In *N. benthamiana*, the silencing of two *EXO70B* paralogues led to increased susceptibility to *Phytophthora infestans* [6]. Three of the 23 members of the *Arabidopsis EXO70* gene family (*EXO70B1*, *EXO70B2* and *EXO70H1*) have been proven to be involved in plant immunity [26]. *AtEXO70B1* and *AtEXO70B2* belong to the same subgroup and are both involved in plant immunity, of which *AtEXO70B1* a negative regulator, while *AtEXO70B2* is a positive regulator. *AtEXO70B1* underwent autophagic transport, and the loss-of-function of *exo70B1* led to reduced numbers of internalized autophagosomes, accumulation of salicylic acid (SA), and finally, ectopic hypersensitive responses and enhanced resistance to several pathogens. AtEXO70B1’s regulation of disease resistance, either by interacting with TIR-NBS2, a truncated version of the classical nucleotide binding (NB) domain and a leucine-rich repeat (LRR)-containing (NLR) intracellular immune receptor-like protein [27,28,29], or by interacting with RIN4, a well-known regulator of pathogen-associated molecular pattern (PAMP)-triggered immunity (PTI) [29]. AtEXO70B2 regulated innate immunity via interacting with a negative PTI regulator, AtPUB22, which mediated the ubiquitination and degradation of AtEXO70B2 and contributed to PTI. The *exo70B2* mutants showed aberrant papillae with halos and were susceptible to different PAMPs and pathogens [30,31]. *AtEXO70B1* and *AtEXO70B2* also contribute to the abiotic stress response; both were positive regulators of stomatal movement. The response to mannitol (drought) treatments is in either an abscisic acid (ABA)-dependent or -independent manner [32,33]. *AtEXO70H1* is a homolog of *AtEXO70B2* and is also involved in plant immunity [31]. Three of the 47 *EXO70* members of rice (*OsEXO70E1*, *OsEXO70F2* and *OsEXO70F3*) were reported to participate in plant immunity [5]. OsEXO70E1 is attributed to planthopper resistance by interacting with a broad resistance protein, *Bph6*. Interaction of the two proteins increased exocytosis and blocked the feeding of a planthopper by cell wall thickening at the infection sites [34]. The importance of *EXO70* in plant immunity was also shown by the fact that some of the *EXO70s* were targets of the secreted effectors of the plant pathogen. Both OsEXO70F2 and OsEXO70F3 were targets of the *Magnaporthe oryzae* effector AVR-Pii, and OsEXO70F3 was proven to play an important role in *Pii*-dependent resistance by interacting with AVR-Pii [35].

Accumulated evidence has shown that a large number of *EXO70s* exist in plants; however, only a few have had their biological roles elucidated [5,35,36]. Due to the huge genome size and complexity [37], the knowledge of the *EXO70* gene family from the *Triticeae* species is rather limited. In the last five years, the genome sequences of wheat and its ancestor species have been released, which makes genome-wide identification of a gene family in the *Triticeae* species feasible [38,39,40,41,42,43]. 

*Haynaldia villosa* L. (2*n* = 2x = 14, VV) is a diploid wild relative of wheat. Previous studies showed that *H. villosa* is a valuable genetic resource harboring many elite traits, such as resistance to several wheat diseases and tolerance to abiotic stresses [44,45,46]. In the present study, different members of the *EXO70* gene family are identified by browsing the released genome sequences of the *Triticea* species. Specific primer pairs are designed, *EXO70s* are cloned from *H. villosa* and their potential functions are elucidated by expression profiles based on in silico analysis and quantitative RT-PCR (qRT-PCR). The obtained results would help us to understand the evolution and diversification of the *EXO70s* among *Triticeae* species and their potential roles in plant immunity and responses to abiotic stresses.

## 2. Results

### 2.1. Identification and Phylogenetic Relationship Analysis of the EXO70 Gene Family in Triticeae Species

**Figure 1:**
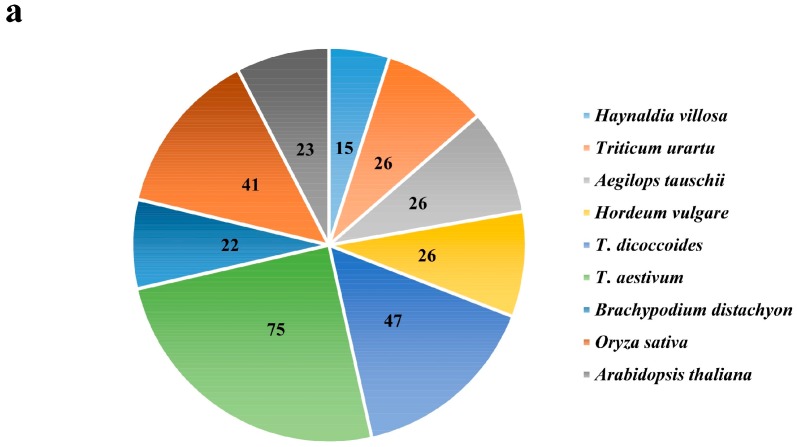

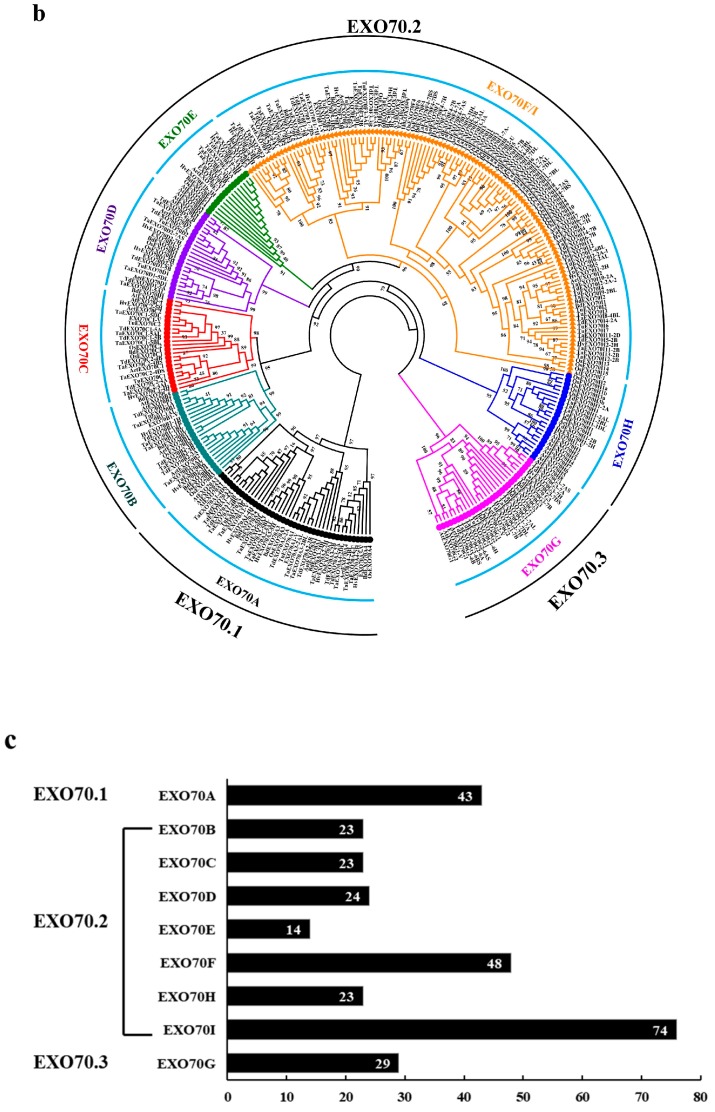
The number and phylogenetic relationships of *EXO70* family genes from *T. aestivum*, *T. urartu*, *Ae. tauschii*, *T. dicoccoides*, *H. vulgare*, *A. thaliana*, *Oryza sativa*, *Brachypodium distachyon* and *H. villosa*. (**a**) The total number of *EXO70* gene family in nine species. (**b**) The phylogenetic tree of nine *Triticeae* species. Species abbreviations: Ta, *Triticum aestivum*; Tu, *Triticum urartu*; Aet, *Aegilops tauschii*; Td, *Triticum dicoccoides*; Hv, *Hordeum vulgare*; At, *Arabidopsis thaliana*; Bd, *Brachypodium distachyon*; Os, *Oryza sativa*; -V, *Haynaldia villosa*. (**c**) The number of the *EXO70* gene family from nine species in each of the subgroups. The horizontal/longitudinal coordinate axis represents the number of genes and different subgroups, respectively.

In total, 200 *EXO70* genes were identified from the public database of five *Triticeae* species. Among them, there were 26 each from *T. urartu*, *Ae. Tauschii* and *H. vulgare*; 47 from *T. dicoccoides*; and 75 from common wheat (*T. aestivum)*, respectively. Fifteen *EXO70s* from *H. villosa* were obtained by homology cloning (Figure 1a). The evolutionary relationship of the above 215 *Triticeae EXO70s*, along with 22 from *Brachypodium distachyon*, 41 from rice and 23 from *Arabidopsis*, were phylogenetically analyzed (Figure 1b, Table S1). These *EXO70s* were divided into three major groups, *EXO70.1*, *EXO70.2* and *EXO70.3*, which were further assigned to nine subgroups, from *EXO70A* to *EXO70I*, according to a phylogenetic tree (Figure 1b). The *EXO70A* subgroup belongs to the group *EXO70.1*, in which 43 (14.28%) *EXO70s* were included; the EXO70B, C, D, E, F, H, and I subgroups belong to the group *EXO70.2*, in which 229 (76.08%) *EXO70s* were included; the *EXO70G* subgroup belongs to the group *EXO70.3*, in which 29 (9.63%) *EXO70s* were included. The *EXO70I* subgroup has the most members (74, 24.58%), followed by *EXO70F* (48, 15.95%) and *EXO70A* (43, 14.28%) (Figure 1c). Based on the subgroups and genome allocation, the wheat *EXO70s* were designated [7,47]. For example, the *EXO70B1* from *T. dicoccoides* located on chromosome 1A was assigned *TdEXO70B1-1A*. In *Arabidopsis*, the *EXO70I* subgroup is missing, whereas the *EXO70I* subgroup in *Triticeae* species appeared to be the most divergent. However, our analysis led to a new insight: the *EXO70I* subgroup belongs to *EXO70.2*, rather than *EXO70.3* from other species [36] (Figure 1b,c).

The 15 cloned *EXO70s* from *H. villosa* were designated as *EXO70A1*-*V* to *EXO70I1*-*V* according to the phylogenetic relationship to wheat *EXO70s*. They belong to nine subgroups, three each to *EXO70A* and *F*, two each to *EXO70D* and *G* and one each to *EXO70B*, *C*, *E*, *H* and *I* (Figure 1). Their CDS length ranges from 801 bp (*EXO70H1-V*) to 2007 bp (*EXO70C1-V*) and their isoelectric point varies from 4.52 (*EXO70B1-V*) to 10.19 (*EXO70G1-V*) (Table 1).

**Figure 2:**
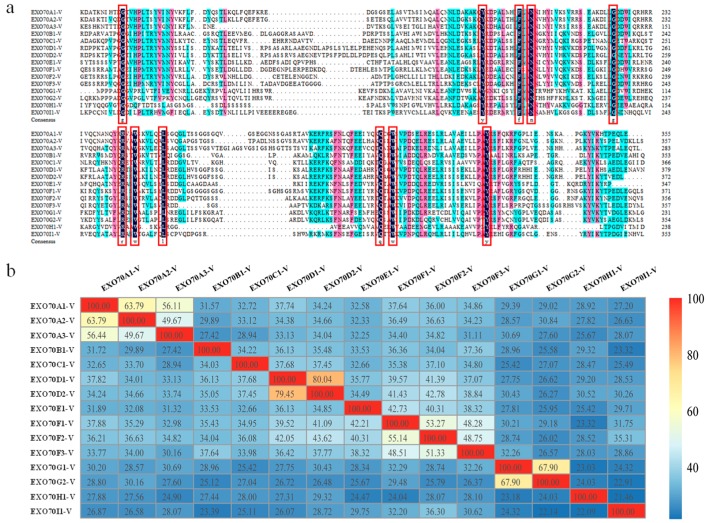
The amino acid sequence feature analysis of *EXO70* gene in *H. villosa*. (**a**) The amino acid sequence of pfam03081 domain for each *EXO70* was selected for multiple sequence alignment analysis by DNAMan. The 11 identical amino acids were indicated in red frame. The three colors of black, red and blue represent the level of similarity of amino acids, from high to low. (**b**) Sequence similarity analysis using the R programming language. The color scale bar represents sequence similarity between different genes. Red and yellow indicate that the sequence similarity was greater than 80% and 60%, respectively. Blue indicates that the sequence similarity was less than 40%.

DNAMan was used to explore the amino acid sequence feature of *H. villosa EXO70s*. The pfam03081 domain at the C-terminus was relatively conservative, shown by the fact that all of the *EXO70s* had the same amino acid in the 11 sites (in the red rectangle in Figure 2a). R programming language was used to visualize the sequence similarities. The same subgroups shared more common sequences, e.g., *EXO70D1-V* and *EXO70D2*-*V* shared about 80% similarity, while different subgroups had low sequence similarities, e.g., *EXO70H1-V* and *EXO70I1-V* only had 21.46% similarity (Figure 2b).

### 2.2. The Number of EXO70s in Genomes of Triticeae Species

The diploid species *B. distachyon* and *Arabidopsis* have 22 and 23 *EXO70s*, respectively, while diploid rice has 41, nearly twice as many. We identified 22 *EXO70s* each in A and D of common wheat and B of the tetraploid wheat, 25 in A of tetraploid wheat, 26 in D of *Ae. Tauschii* and H of barley and 29 *EXO70s* in B of common wheat. For the A genome, tetraploid has three more *EXO70s* than hexaploidy wheat; for the B genome, hexaploidy has seven more than tetraploid wheat; for the D genome, *Ae. tauschii* has four more *EXO70s* than hexaploidy wheat. In common wheat, the chromosomes 5A, 5B and 5D had the same number of *EXO70s*, belonging to the same gene type and having the same gene order. The chromosomes 7A, 7B and 7D also have the same number of *EXO70s*; however, their gene type and gene order were not completely the same. For example, *TaEXO70D* is only present on 7A and 7B, but not on 7D; *TaEXO70G1* is only present on 7A and 7D, but not 7B. The gene number and order for *EXO70s* in the remaining five homoeologous were also different among corresponding homoeologous chromosomes, mainly due to their difference for subgroup *EXO70I* (Table 2 and Table S2).

The three analyzed diploid *Triticea* species, *H. vulgare*, *T. uratu* and *Ae. tauschii*, each had 26 *EXO70s* and had the same number as the *EXO70B*, *C*, *D* and H subgroups. They had the least *EXO70Hs* and the most *EXO70Is*. *T. uratu* only had one *EXO70G*, while the other two species had three. However, *T. uratu* had more *EXO70I* than the other two species (Table 3). The variation among different diploid species may be due either to the quality of the genome assembly or the duplication or deletion during evolution. The tetraploid *T. dicoccoides* and hexaploid *T. aestivum* have 47 and 75 *EXO70s*, which are about twice and three times the number in diploid species, respectively. In addition, the number of *EXO70s* from each subgroup is also exactly (*EXO70C*, *H*) or almost (*EXO70A*, *B*, *D*, *E*, *F*, *I*) 2 or 3 times that of the diploid species (Table 3). This indicated that, in polyploid wheat, the increased number of *EXO70s* is mostly due to the genome polyploidization.

We only identified 15 *EXO70s* in diploid *H. villosa*, which is much less than the average number for other diploid species. *H. villosa* had the same number of subgroups D and H, but fewer members of subgroups B, C, E, F and I. This is due either to the lack of the sequence of *H. villosa*, or the divergence of *H. villosa* from other *Triticea* species (Table 3).

### 2.3. The Chromosomal Distribution of EXO70s in Triticeae Species

**Figure 3:**
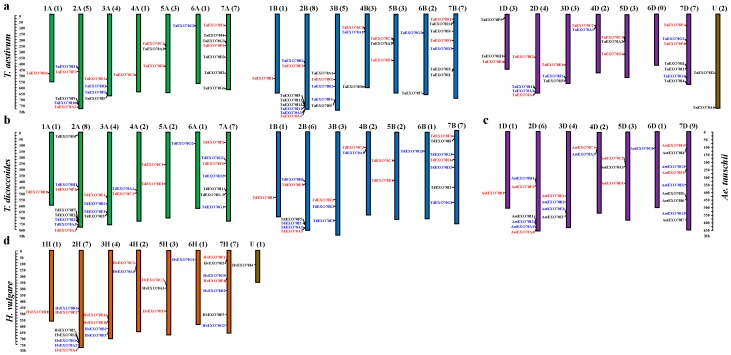
The chromosomal distribution of the *EXO70* gene family in four *Triticeae* species. (**a**–**d**) represent *T. aestivum*, *T. dicoccoides*, *A. tauschii* and *H. vulgare*, respectively. Chromosome numbers are indicated at the top of each bar and the number in parentheses corresponds to the number of *EXO70* genes present on that chromosome. The name of each gene is to the left of each chromosome. Gene names labeled with red, blue, or black indicate that they are conserved in four species, missing in one due to incomplete data, or missing in more than two, respectively.

The identified 174 *EXO70s* from four *Triticeae* species (*T. aestivum*, *Ae. tauschii*, *T. dicoccoides* and *H. vulgare*) were assigned to corresponding chromosomes (including three on unknown chromosome). With the exception of wheat 6D, all chromosomes have at least one *EXO70* gene (Figure 3). The *EXO70s* were not evenly distributed on difference chromosomes or in different homologous groups. In total, there were 9, 44, 27, 14, 19, 7 and 51 *EXO70s* in the homologous groups 1 to 7, with group 7 having the most *EXO70s* (29.31%), followed by group 2 (25.29%) (Table 2). The *EXO70s* in group 7 was dispersely distributed along the chromosome, while those in group 2 were clutered at the dital region of the long arm (Figure 3). We found that nine groups *EXO70s* were conversely present on the same homoeologous groups of the four species, such as the B1 in group1, F2/3 in group 2, E1 and F3/2 in group 3, C2/C1 in group 4, CI and D1 in group 5, F1 and F4 in group 7 (Table S2, Figure 3). 

For homologous chromosomes from different genomes of wheat and its ancestral/related species, most of the corresponding *EXO70* orthologs were present at the syntenic genome regions. For example, *EXO70B1s* were on the long chromosome arm of homoeologous group 1 and *EXO70E1* on group 3 chromosomes (Figure 3). There are some exceptions. The *EXO70G1* is present on all group 6 chromosomes except for sub-genome 6D of the hexaploidy wheat. The *EXO70H1* is located on chromosomes 2A, 2B of common wheat and *T. dicoccoides*, 2D of *Ae. tauschii* and 2H of barley and on 1D of common wheat (Figure 3). We found that the *EXO70C2* is on the 4BS of common wheat and *T. dicoccoides* (Figure 3a,b), on the 4DS of common wheat and *Ae. tauschii* (Figure 3a,d), but on the 4AL of common wheat and *T. dicoccoides* (Figure 3a,b). This also supports the presence of an inter-arm translocation of 4A during the evolution from diploid to tetraploid wheat [48,49]. 

### 2.4. The Diversification of Gene Structure of Triticeae EXO70s

The C-terminal PFam03081 domain, which may determine the function or structure of the proteins, is a specific characteristic of the *EXO70* superfamily [47]. All the predicted 200 and 15 homologous cloned EXO70 proteins possessed such a domain; however, their amino acid sequence length is different for different *EXO70s*, varying from 103aa to 669aa and with an average length of 345aa (Table S1).

**Figure 4:**
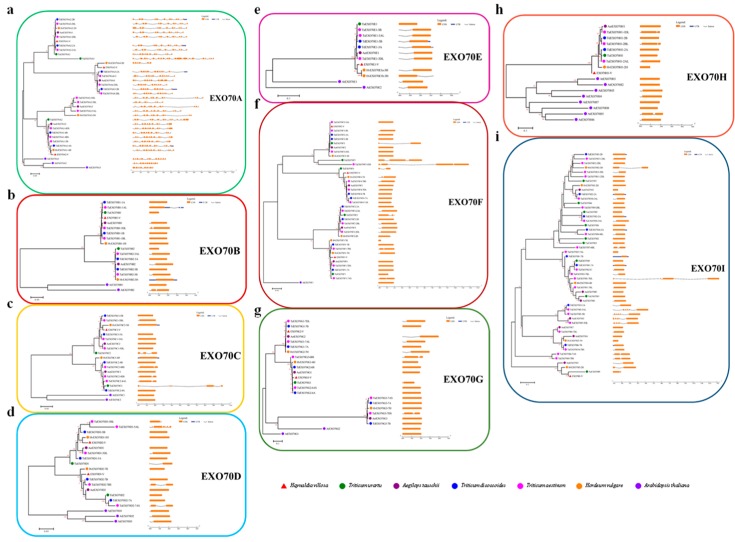
Phylogenetic analysis and exon–intron structures of *EXO70* gene family in common wheat and related *Triticeae* species. The phylogenetic analysis was performed using the sequences of the conserved domain of *EXO70*; proteins were aligned by ClustalW, constructed by MEGA6 using the N-J method, with 1000 bootstrap replicates; the branch length scale bar indicates the evolutionary distance. The left column identifies subgroups and is marked with different alternating background tones to make subgroup identification easier. Introns and exons are represented by black lines and colored boxes, respectively.

The exon–intron structure of the 200 *Triticeae EXO70s* was visualized by using the online Gene Structure Display Server and compared among different subgroups or within each individual subgroup. The exon-intron structures for most of the *EXO70s* within the same subgroups were relatively conserved among *Triticeae* species, was similar to in *Arabidopsis* or rice [7,36]. Compared with other subgroups, the subgroup *EXO70A* with 30 genes has the most introns on average, e.g., *TuEXO70A3* has the most introns (20) and *HvEXO70A4-2H* has the fewest introns (five) (Figure 4a). The 170 *EXO70* genes in the remaining eight subgroups only have about one intron on average. Among them, 83 genes (41.50%) are intronless, including eight *EXO70Bs*, seven *EXO70Cs*, nine *EXO70Ds*, one *EXO70Es*, 18 *EXO70sF*, 12 *EXO70Gs*, five *EXO70Hs* and 23 *EXO70sI*; 60 genes (30.00%) have one intron (e.g., *AetEXO70B1* and *TaEXO70C1-5BL*), 19 genes (9.50%) have two introns (e.g., *TuEXO70C2* and *TaEXO70G2-6BS*) and eight genes (4.00%) have three to six introns (e.g., *AetEXO70I3* and *TaEXO70D1-5AL*). We also observed that genes that have a closer phylogenetic relationship in the same subgroup have a similar gene structure; however, within the same subgroup, some genes showed quite different gene structure. For instance, *TaEXO70D1-5AL* has six introns in *EXO70D* (Figure 4d) and the three copies of *TaEXO70I5* and *AetEXO70I3* have five introns, while the other genes in the same subgroup (*EXO70D* or *EXO70I*) only have one or two introns (Figure 4i). The most diversified gene structure of the *EXO70A* subgroup may be a clue that their diversified biological role is different from that of other subgroups.

### 2.5. The Expression Pattern of TaEXO70 Genes

**Figure 5:**
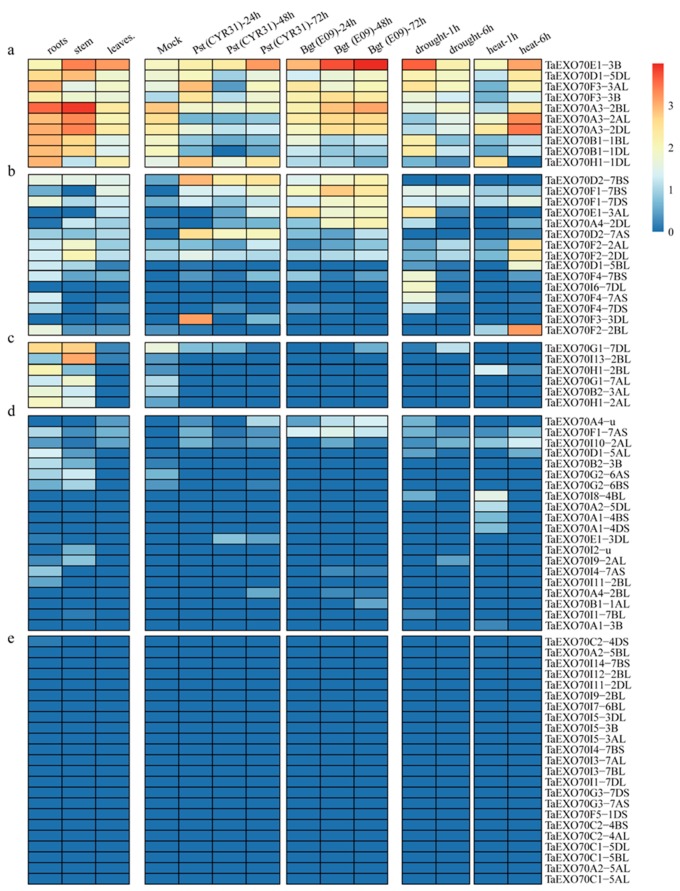
Heat map of the expression profiling of wheat *EXO70* genes in different tissues and under various stresses. The color scale bar represents the expression values of the genes. (**a**–**e**): Genes with different expression types. Abbreviations: *Bgt*, powdery mildew; *Pst*: Stripe rust.

The expression patterns of different members in a gene family will help us predict their potential biological roles. To elucidate the potential roles of the identified *EXO70s*, their expression in different tissues or in responses to various biotic and abiotic stresses was investigated by in silico expression profiling or qRT-PCR analysis. The expression patterns of wheat *TaEXO70s* in different tissues (root, stem and leaf of seeding stage), under two biotic stresses (stripe rust pathogen *CYR31* and powdery mildew pathogen *E09*) and two abiotic stresses (drought and heat) [50,51] were first investigated using the wheat RNA-seq data from the publicly available databases. The expression level was measured as tags per million (TPM). To facilitate the portraits of transcript abundance, we assume the expression was high if TPM ≥ 2.5; moderate if 2.5 > TPM ≥ 1.5; low if 1.5 >TPM > 0; and undetectable if TPM = 0. All 75 *TaEXO70* genes exhibited significantly diverse expression patterns (Figure 5). Their expression can be classified into five groups. The group “e” includes 23 *TaEXO70s* (30.67%). Their transcript abundance was undetectable; among them 11 were from subgroup I and six were from subgroup C (Figure 5e). The group “d” includes 20 genes (26.67%). They had detectable but weak transcript abundance; among them six were from subgroup A and six were from subgroup I (Figure 5d). The group “c” has six genes (8.00%), whose expression is high in roots and stems, whereas it does not respond to the four stresses (Figure 5c). The group “b” has 15 genes (20.00%). These genes were found to be negligibly to moderately expressed in the three tissue types and in response to one or two stresses. Among them, nine were from the subgroup F (Figure 5b). The group “a” has ten genes (13.33%). Their expression was generally high, either in different tissues or in response to the four stresses and none of them were from the subgroup I (Figure 5a).

Six genes were upregulated in response to both biotic and abiotic stresses. *TaEXO70E1-3B* was the only gene that was highly expressed in three tissues and upregulated in response to all four stresses. Its homologs on 3AL *TaEXO70E1-3AL* displayed moderate expression in three organs and were only responsive to *Bgt* inoculation and drought stress. The *TaEXO70D1-5DL* and *TaEXO70F3-3B* also displayed moderate expression in three organs and stresses. The *TaEXO70H1-1DL* expression was higher in root/leaf tissue and showed upregulation under both *Pst* infection and heat treatment. The *TaEXO70B1-1BL/1DL* expression was higher in root/stem tissue, and it was downregulated in response to *Bgt* and *Pst* inoculation but upregulated by drought treatment. *TaEXO70A3-2AL/2BL/2DL* had a similar expression pattern, being expressed in three tissues (stems > roots > leaves) and downregulated by *Pst* inoculation but upregulated by both drought and heat treatment. *TaEXO70D2-7BS* and *TaEXO70G1-7DL* were both only responsive to biotic stresses; however, they showed opposite patterns, with *TaEXO70D2-7BS* upregulated and *TaEXO70G1-7DL* downregulated. Twelve genes were only responsive to one of the four stresses; five were only upregulated in response to *Bgt* or *Pst* inoculation; and seven were only upregulated in response to drought or heat stress. Conservation roles were observed for all three homolog genes from different genomes, such as *TaEXO70A3(2AL/2BL/2DL)* and *TaEXO70F2(2AL/2BL/2DL)*.

### 2.6. Differential Expression of 15 EXO70 Genes from H. villosa

**Figure 6:**
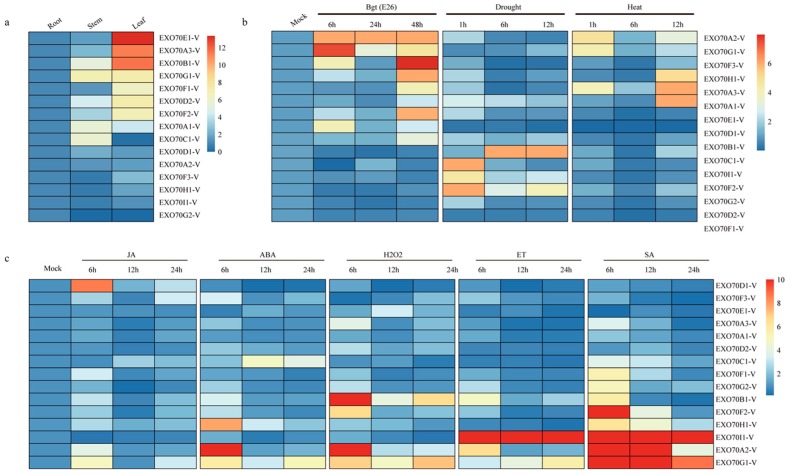
Heat map of the expression profiling of wheat and *H. villosa EXO70* genes in tissues and in response to biotic/abiotic stress, phytohormones and H_2_O_2_ treatments. (**a**) Tissue-specific expression pattern of 15 *EXO70-V* genes in *H. villosa*. (**b**) Expression levels of *EXO70-V* genes in biotic/abiotic stresses of *H. villosa*. (**c**) Expression profiling of *EXO70-V* genes in response to phytohormones and H_2_O_2_ treatments. The scale bar showing expression level of the genes. Abbreviations: *Bgt*, powdery mildew; SA, salicylic acid; MeJA, methyl jasmonate; ET, ethephon; ABA, abscisic acid; H_2_O_2_, hydrogen peroxide.

The expression patterns of 15 *EXO70*-Vs in different tissues and in response to different stresses or treatments in *H. villosa* were investigated by qRT-PCR. Different expression patterns were observed for the analyzed genes (Figure 6). Six of the genes (*EXO70D1-V*, *EXO70A2-V*, *EXO70F3-V*, *EXO70H1*-V, *EXO70I1*-V and *EXO70G2*-V) were not differentially expressed in all three tissues. Four genes (*EXO70B1*-V, *EXO70G1*-V, *EXO70A1*-V, *EXO70C1*-V) showed more abundant transcript in stem; seven genes (*EXO70E1*-V, *EXO70A3*-V, *EXO70B1*-V, *EXO70G1*-V, *EXO70F1*-V, *EXO70D2*-V and *EXO70F2*-V) showed higher expression level in leaves (Figure 6a).

*EXO70A2-V* showed a similar expression level in all the three tissues; however, its expression was significantly increased in response to *Bgt* inoculation and treatments by chitin, flg22, heat stress, phytohormones, or H_2_O_2_. *EXO70G1-V* showed a similar expression level in the stems and leaves; its expression was also significantly increased by *Bgt* inoculation and treatment by chitin, flg22, four phytohormones, or H_2_O_2_. The expression of *EXO70H1-V* was strongly upregulated by *Bgt* inoculation and cold stress, and moderately upregulated by chitin, Flg22, ET and heat stress. *EXO70I1-V* only responded to abiotic stresses (drought, salt and cold) and ABA treatment. *EXOG2-V* was responsive to flg22 treatment and drought stress, and *EXO70A3-V* was only responsive to heat stress (Figure 6b,c). The divergence of expression patterns of different gene members indicated their clear-cut roles in the adaptation of *H. villosa* to various environments or stresses.

### 2.7. The Subcellular Localization of EXO70s from H. villosa

**Figure 7:**
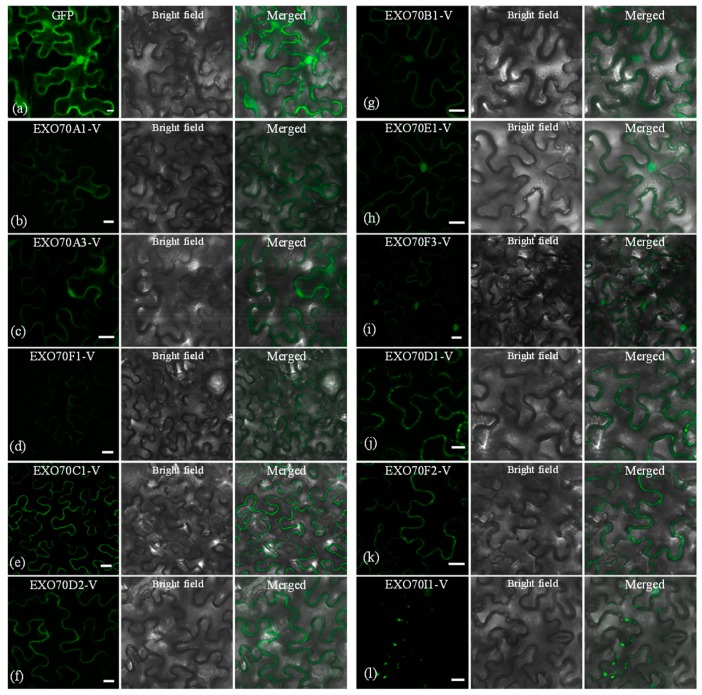
Subcellular localization of *H. villosa* EXO70-GFP-Vs proteins. *H. villosa* EXO70-GFP-Vs proteins were transiently expressed in *N. benthamiana* leaves carried out with injection of *Agrobacterium* and examined by a confocal microscope. Green fluorescence was observed 48 h after infection. Bar = 20 μm. (**a**) The empty GFP vector was used as the control. The green channel shows that GFP signals were localized in the nucleus and cytoplasmic and plasma membranes. (**b**–**l**) The subcellular localization pattern of EXO70A1-V to EXO70I1-V, respectively.

Knowledge of the subcellular localization of a plant protein can help us predict its potential role in the biological process. The subcellular localization of EXO70-Vs was investigated by transiently expressing the construct into leaves of *Nicotiana tabacum* via *Agrobacterium* method. Eleven EXO70-Vs generated fluorescence signals. Compared with the relatively even distribution of GFP signals in the cell (Figure 7a), the 11 confusion proteins had distinct localization patterns. EXO70A1, A3 and F1-V displayed weak signals on the plasma membrane (PM) (Figure 7b–d), while the PM signals for EXO70C1-V and EXO70D2-V were more intensive (Figure 7e,f). EXO70B1, E1 and F3-V displayed signals both in the PM and the nucleus (Figure 7g–i). EXO70D1-V and EXO70F2-V also produced signals in the PM; in addition, they also had small and discrete spot signals in the PM (Figure 7j,k). EXO70I1-V was the only one with no continuous PM localized signal; however, we observed discrete punctate signals along the PM (Figure 7l).

## 3. Discussion

### 3.1. Evolutionary Relationship of the EXO70 Gene Family in Wheat and Its Relatives

The evolutionary relationships of the *EXO70* gene family (between wheat, *T. urartu, Ae. tauschii, T. dicoccoides, H. vulgare* and *H. villosa*) have been speculated about based on the total number, classification, chromosomal distribution and structure. The surveyed diploid species of seven chromosome pairs except for *H. villosa* all possessed 26 *EXO70* genes, which suggested this gene family appeared before the divergence among Triticum species [52]. Allohexaploid wheat originated from two hybridizations between three diploid progenitors approximately 2.5–4.5 million years ago [53]. The number of *EXO70* genes in tetraploid and common wheat (a total of 47 and 75, respectively) is approximately twice and three times as many as diploids, implying they have undergone one and two rounds of polyploidization events [54]. Although the polyploidization event induced rapid and extensive genetic and epigenetic changes in the genome which were related to a large range of molecular and physiological adjustment [55] as well as a significant loss of gene family members upon domestication [53], by comparing with diploid species, the *EXO70* gene family did not go through a wide range of expansion or diminution in tetraploid wheat and allohexaploid wheat. This deduction is also supported by their chromosomal location analysis, which showed that 73.1% of genes have good collinearity among wheat, *Ae. tauschii, T. dicoccoides* and *H. vulgare*, and most of the same type of orthologous genes maintain the relative order of their ancestral genes (Figure 3 and Figure 4). As for the small difference in the number of genes on individual subgroups among wheat and its relatives, this may be because of the quality of the genome assembling or the gene duplication of subgroup *EXO70I*.

Phylogenetic analysis showed that all three groups (*EXO70.1*, *EXO70.2* and *EXO70.3*) and nine subgroups (*EXO70A* to *EXO70I*) are represented in each of the six *Triticeae* species. A similar gene structure was found in the same subgroup; subgroup *EXO70A* consists of multiple introns, while the other eight subgroups had fewer or were intronless. The variable intron numbers confirmed the classifications of the *EXO70* genes. Additionally, *EXO70* subgroups diversified before the divergence within polyploid wheat and related species during the evolutionary process of the *EXO70* gene family; however, no new groups/subgroups have emerged. 

*EXO70I* members were most represented in wheat and its five relatives (57), as well as in rice (16), but not in *Arabidopsis* [6,36]. *EXO70I* belongs to *EXO70.2*, not *EXO70.3*, which is different from what was found in previous studies [1,7,36]. This suggests that the *EXO70I* subgroup arose before the evolutionary divergence of rice from other *Triticeae* crops and disappeared during the evolution of *Arabidopsis*. The *EXO70I* subgroup underwent rapid divergence, producing a large number of members; this event can probably be explained by unequal cross-over or segmental chromosomal duplication [56,57]. During long-term natural selection, numerous *EXO70* genes diverged and evolved in order to respond to various conditions. The study of *Arabidopsis* showed that the duplicated gene loss process is non-random; those involved in DNA repair are more likely to be lost, while genes involved in signal transduction and transcription have been preferentially retained [56]. Therefore, the function of *EXO70I* subgroup in *Arabidopsis* was inclined to responses to DNA repair, and in grass species may participate in signal transduction. Research on a larger range of species is needed to figure out whether the *EXO70I* branch is unique to monocotyledons.

### 3.2. Diversification of Subcellular Localization Pattern of the EXO70 in H. villosa 

Protein subcellular localization analysis provided important clues to their specialized biological functions [58]. The diversification of EXO70-V subcellular localization patterns implies functional differentiation. Except for EXO70I1-V, all *EXO70* genes showed plasma membrane (PM) signals. The EXO70D1/F2-V-GFP locates to the PM merged with some small, discrete punctate. At the same time, EXO70I1-V-GFP only gave rise to smaller fluorescent discrete punctate along with PM, which are similar to AtEXO70E2 in *Arabidopsis* protoplasts, which was a maker of a novel double-membraned structure termed EXPO (exocyst-positive organelles). AtEXO70E2 was involved in unconventional protein secretion for cytosolic proteins that lack a signal peptide, because of its ability to recruit several other exocyst complex subunits [8,9,59,60]. Therefore, *EXO70D1*, *F2* and *I1-V* might have the ability to recruit different partners, then form various complexes to execute different biological functions. AtEXO70A1 was distributed in different patterns in different systems’ cytosol; it showed up in the nucleus and numerous small punctate structures in the BY-2 cell [36], at the apex of growing tobacco pollen tubes [61] and is strongly present in the cell plate [62]. In the study, EXO70A1/A3-V showed a weak PM signal, while EXO70A2-V was characterized by mis-localizations. This was probably because *AtEXO70A1* took part in a different vesicular transport process. Moreover, despite both AtEXO70B2 and AtEXO70H1 participating in the interaction between plants and pathogens, the signal of EXO70B2-GFP was mainly found in the cytoplasm, while EXO70H1-GFP was in vesicle-like structures in *Nicotiana benthamiana* leaf [31]. In our analysis, EXO70B1-V was present in the PM and nucleus (Figure 7g). It is likely that they went through different action sites to take part in the process of disease resistance. An exocyst is a tethering factor that mediates secretory vesicles to the plasma membrane before SNARE-mediated fusion [63,64]. EXPOs deliver cytosolic proteins to the cell surface [65,66] and therefore all of those were related to PM. The results explained why most genes had the PM location pattern.

### 3.3. Function Conserve or Differentiation of the EXO70 Gene Family in Common Wheat and H. villosa 

An orthologous gene is one that diverged after evolution to give rise to different species; this gene generally maintains a similar function to that of the ancestral gene that it evolved from [67]. In our study, some *EXO70* orthologous genes from *H. villosa* exhibited a similar expression pattern to common wheat. For example, *EXO70A3-V* and *TaEXO70A3* showed a high expression level under heat stress; *EXO70B1-V* was preferred to *TaEXO70B1-3AL*, which was induced by *Bgt* at a late stage (48 h), but not by drought and heat stress; *EXO70E1-V* and *TaEXO70E1-3B* were upregulated by *Bgt* treatment; the expression of *EXO70H1-V* and *TaEXO70H1-1DL* was increased in response to heat (Figure 5 and Figure 6). Therefore, it is reasonable to presume that some *EXO70* genes from *H. villosa* may have a similar function to the corresponding *EXO70* genes from common wheat. In plants, *AtEXO70B1*, *AtEXO70B2*, *AtEXO70H1* and *OsEXO70E1* are known for their roles in innate immunity [6,27,28,29,30,31,34]. In the literature, genes such as *TaEXO70B1*/*B2* (with their homologous alleles), *EXO70B1-V*, *TaEXO70E1-3B*, *EXO70E1-V*, *TaEXO70H1-1DL* and *EXO70H1-V* were induced by *Pst*/*Bgt* treatment. Thus, we hypothesize that those genes also play an important role in plant defense responses and it is worth conducting further study to prove their function.

The long-term evolutionary fate of paralogous genes will still be determined by functions, with the genes that appeared to be sub-functionalized or neo-functionalized probably having higher rates of gene birth because of the increased adaptability. In contrast, the functional redundancy gene is unlikely to be stably maintained in the genome [55,57,68,69]. Paralogous/orthologous genes may diverge in expression to achieve more complex control of the same genetic network, balancing the relationship between internal growth and external environmental stimuli so that they “pay” the least and get the most [70]. In *Arabidopsis*, *EXO70C1*/C2 were involved in pollen development and mainly localized pollen related tissue [21,22]. In common wheat, six *TaEXO70C* genes had an undetectable expression level in roots/stem/leaves (Figure 5a), but *EXO70C1-V* showed a moderate expression level in the stem and responded to drought and ABA treatment (Figure 6). Studies have shown that ABA has contributed to osmotic stress tolerance by regulating stomatal aperture and guard cells [71]. Therefore, *EXO70C1-V* perhaps plays an important role in drought tolerance. In rice, OsExo70F3 interacts with AVR-Pii and plays a crucial role in triggered immunity [35]. In our research, including the copy number, 13 *TaEXO70F* members were identified and *EXO70F1-V*, *EXO70F2-V* and *EXO70F3-V* were cloned from *H. villosa*. Expression studies have revealed that 11/13 common wheat varieties were induced by stress treatment (Figure 5), and three *EXO70-V* genes showed clearly diverse expression patterns, of which *EXO70F1-V* was only induced by SA, *EXO70F2-V* in response to drought, H_2_O_2_ and SA at an early stage, and *EXO70F3-V* in response to *Bgt* treatment at a late stage (48 h) (Figure 6). Research shows that both SA and H_2_O_2_ function as a key regulator against pathogens and stress tolerance [72,73,74]. Thus, we can infer the function of *EXO70F* genes not only in plant defense responses but also in abiotic stress. 

In *N. benthamiana*, *EXO70D* and *EXO70G* mainly affect the size of the leaf [6]. In wheat, *TaEXO70D2-7BS* and *TaEXO70G1/G2* (with their homeoalleles) were induced by *Pst* and *Bgt* treatments. *EXO70D1-V* was upregulated by *Bgt* and MeJA treatments, *EXO70G1-V* had an increased expression level under phytohormones and H_2_O_2_ treatments, while *EXO70G2-V* was induced by drought and SA. MeJA is important for regulating the growth of plants and promotes plant resistance of various stresses [75]. This might suggest that *EXO70D1-V* plays an important role in plant growth and defense responses *EXO70G* are multifunctional. Of 22 *EXO70I* genes from common wheat, only five genes had distinct inducible expression. For instance, *TaEXO70I6-7DL* and *TaEXO70I8-4BL* were upregulated by drought and heat, respectively. *EXO70I1-V* was induced by ET and SA and maintained a high expression level. ET and SA regulate many diverse metabolic and developmental processes in plants, such as seed germination, abiotic stress response and pathogen defense [76,77]. Thus, we hypothesize that *EXO70I1-V* might play a vital role in growth or against multiple stresses. *EXO70* genes provide diverse expression patterns in different tissues and stresses, implying that *EXO70* genes may play an essential role in plant adaptation to a complicated and changeable environment.

## 4. Materials and Methods 

### 4.1. Plant Materials 

*H. villosa* (genome VV, accession no. 91C43), from the Cambridge Botanical Garden, Cambridge, UK, was used for gene cloning and expression analysis. Powdery mildew susceptible variety Sumai 3 was used for propagation of fresh spores of powdery mildew isolate *E26*. *Nicotiana benthamiana* plants were used for subcellular localization analysis. All the materials were grown in a greenhouse under a 14 h light/10 h dark cycle at 24 °C/18 °C, with 70% relative air humidity.

### 4.2. Plant Treatments

The seedlings of *H. villosa* were grown in liquid or soil until the three-leaf stage. For heat shock or drought stress treatment, the plants were transferred to 42 °C conditions, or dipped into 20% PEG 6000 and leaves were sampled at 0, 1, 6, or 12 h after treatment. For powdery mildew treatment, the plant was inoculated with pathogen isolate *E26* and the leaf tissues were sampled at 0, 24, 48 and 72 h after inoculation. For phytohormones and H_2_O_2_ treatments, the plants were sprayed with 5 mmol salicylic acid (SA), 0.1 mmol methyl jasmonate (MeJA), 0.1 mmol ethephon (ET), 0.2 mmol abscisic acid (ABA) and 7 mmol hydrogen peroxide (H_2_O_2_), respectively and all leaf tissues were collected at 0, 6, 12, or 24 h after spraying [78]. All the samples were rapidly frozen in liquid nitrogen, then stored in an ultra-freezer (−80 °C) before use.

### 4.3. RNA Isolation and Real-Time PCR Analysis

Total RNA was extracted using a Trizol Reagent kit (Invitrogen, CA, USA) according to the manufacturer’s instructions and analyzed by gel electrophoresis. The first-strand cDNA was synthesized with random oligonucleotides using the HiScript^®^ II Reverse Transcriptase system (Vazyme, Nanjing, China). qRT-PCR was carried out in a total volume of 20 μL containing 2 μL of cDNA, 0.4 μL gene-specific primers (10 μM), 10 μL SYBR Green Mix and 7.2 μL of RNase free ddH_2_O, using the Roche LightCycler480 Real-time System (Roche, Basel, Swiss Confederation). The expression was represented in the form of relative fold change using the 2^−ΔΔ*C*T^ method [79]. Differentially expressed genes between each two samples pair were defined as two-fold up-regulated or two-fold down-regulated genes. Primers used for qRT-PCR are designed by Primer3 (Table S3). Three biological replications were performed. Heat map analysis of the expression data was performed using heat map drawing software MeV (version No. 4.7, Institute for Genomic Research, MD, USA) 

### 4.4. Identification of EXO70 Gene Families in Wheat and Related Triticeae Species

We searched for the keywords ‘exocyst subunit exo70 family protein’ in the annotated proteins database of *Hordeum vulgare* (HH, 2*n* = 2x = 14, accession No. FJWB02000000) and obtained entries containing gene ID and protein sequences [42]. Then we performed a Conserved Domains (CD) search (https://www.ncbi.nlm.nih.gov/Structure/cdd/wrpsb.cgi) and reserved the protein sequences that contain the typical pfam03081 domain [47]. 

The identified EXO70 protein sequences of *H. vulgare* were used as query sequences to blast (*E*-value ≤ 10^−10^) against protein database of the other species, including *Triticum urartu* (AA, 2*n* = 2x = 14, accession No. NMPL02000000) [39], *T. aestivum* (AABBDD, 2*n* = 6x = 42, accession No. NMPL02000000) [80], *Brachypodium distachyon* (BdBd, 2*n* = 2x = 14, accession No. ADDN03000000) [81], Oryza sativa Japonica (2*n* = 2x = 24, accession No. AP008207–AP008218) [82], *T. dicoccoides* (AABB, 2*n* = 4x = 28, accession No. FXXJ01000000) [43] and *Aegilops tauschii* (DD, 2*n* = 2x = 14, accession No. AOCO02000000) [83]. After removing the redundant gene sequences for each the species, the alignment hits were validated by performing a CD search as described above.

### 4.5. Cloning and Protein Sequences Analysis of EXO70 Genes from Haynaldia villosa

According to the sequences obtained from the database of *H. vulgare*, primers (Table S3) for cloning the full-length cDNA of the *EXO70* gene from *Haynaldia villosa* were designed with online software Primer3 designing tool (v. 0.4.0, University of California, USA) [84] (Table S3). Mixed root, stem and leaf tissue cDNA of *H. villosa* served as a template for the isolation. This was performed at 95 °C for 30 s, followed by 35 cycles of 95 °C for 15 s, 58 °C for 15 s or 30 s and 72 °C for 3 min and then by 5 min at 72 °C in Phanta Max Super-Fidelity DNA polymerase (Vazyme, Nanjing, China). Before subcloning into their destination vectors, the PCR-amplified cDNA products were first cloned into the *pTOPO-Blunt* Vector (Aidlab, Beijing, China) as per the manufacturer’s instructions. Multiple sequence alignments were conducted using DNAMan (Lynnon Corporation, Quebec, QC, Canada) software. The sequences similarity was visualized using the R programming language.

### 4.6. Subcellular Localization Assay

The ORFs of *EXO70-V* genes (without stop codon) were amplified from the *pTOPO-Blunt* Vector, then inserted into the *pCambia1305-GFP* vector, which contains a green fluorescent protein (GFP) reporter gene driven by the CaMV 35S promoter, using homologous cloning technology as per the manufacturer’s instructions (Vazyme, Nanjing, China) (Table S3). Then it was introduced into *Agrobacterium tumefaciens* (strain GV3101) bacteria by a freeze–thaw procedure and grown in Luria-Bertani (LB) medium at 28 °C for 2 or 3 d.

*Agrobacterium tumefaciens* (strain GV3101) bacteria containing fusion constructed were grown in Luria-Bertani (LB) medium with both rifampicin and kanamycin (0.05 µg/mL) at 28 °C overnight. The bacterial cells were centrifuged and resuspended in an infiltration solution (10 mM MES pH 5.6, 0.1 mM Acetosyringone, 10 mM MgCl_2_) to a final OD_600_ = 1.5. Bacterial suspensions were infiltrated into five- to six-week growing stage leaves of *N. benthamiana* by depressing the plunger of a 1-mL disposable needleless syringe into the abaxial side of leaves [85,86]. The fluorescence signals were observed 48–60 h after injection and images were captured using a confocal laser scanning microscope (LSM780; Carl Zeiss, Jena, Germany) according to the methods described by Wang et al. [87].

### 4.7. Phylogenetic Analysis of EXO70 Gene Family

Multiple sequence alignment was conducted by ClustalW which was integrated in Mega v6.0 [88]. Phylogenetic analysis was performed through online software PhyML 3.0 [89] using maximum-likelihood method with default parameter [90]. EXO70 proteins are rather diverse at their N-terminal and could not be aligned reliably, so we only used the conserved domain proteins to construct the phylogenetic tree and removed five genes where the length of the domain is fewer than 200 amino acids for further analysis.

### 4.8. Chromosomal Distribution and Exon-Intron Structure Analysis

Chromosomal information of predicted *EXO70* genes from each species was obtained after using cDNA sequences as a query sequence blasted to the genomic sequence to determine their chromosomal locations. Then we drew their locations onto the physical map of each chromosome using MapInspect tool (http://mapinspect.software.informer.com/). 

The gff3 files of each species was downloaded from the Ensembl Plants FTP server (http://plants.ensembl.org/index.html) on 20 January 2018 for exon–intron structure analysis; the image of the exon–intron structure was obtained using the online Gene Structure Display Server (last accessed date on 20 January 2018) with the gff3 files for each species [91]. The corresponding evolutionary tree were constructed by Mega v6.0 [88], all sequences were aligned by ClustalW using the default parameters [92,93], used the Neighbor-Joining method with the pairwise deletion option, Poisson correction and bootstrap analysis conducted with 1000 replicates [26,94] 

### 4.9. RNA-seq Expression Analysis

Publicly available RNA-seq data were retrieved from the expVIP [50] were used to analyze the expression pattern of predicted wheat *EXO70* genes in different tissues and stresses. The tissue-specific expression data were compiled from three wheat tissues (leaf, stem, root) collected from Chinese Spring at seeding development. The biotic stress expression data included two diseases (stripe rust and powdery mildew pathogen) and were collected from disease-resistant wheat varieties N9134 (at 7 days seedling stage). The abiotic stress expression data, including drought and heat treatments, were collected from heat-resistant wheat cultivar TAM107 (at 7 days seedling stage). The relative expression of each *TaEXO70* gene in different tissues and stresses was presented as a heat map, which was constructed by the heat map drawing software MeV (version No. 4.7, Institute for Genomic Research, MD, USA).

## Figures and Tables

**Table 1:** *EXO70* genes cloned from *H. villosa*.

Number	Name	ORF (bp)	AA (aa)	DL (aa)	PI	MW (KD)
1	EXO70A1-V	1914	637	268–623	7.71	71.48
2	EXO70A2-V	1944	647	273–631	8.78	73.07
3	EXO70A3-V	882	294	1–283	8.83	32.61
4	EXO70B1-V	1626	541	180–534	4.52	59.74
5	EXO70C1-V	2007	668	287–654	5.72	73.9
6	EXO70D1-V	1650	549	152–532	6.98	61.2
7	EXO70D2-V	1644	547	152–525	7.00	61.02
8	EXO70E1-V	1815	605	233–580	5.04	68.93
9	EXO70F1-V	1323	441	52–424	5.46	49.88
10	EXO70F2-V	1542	514	147–504	5.03	57.62
11	EXO70F3-V	1431	477	108–466	5.60	52.91
12	EXO70G1-V	807	268	1–232	10.19	30.95
13	EXO70G2-V	1437	478	85–439	9.42	53.8
14	EXO70H1-V	801	268	1–238	8.66	28.65
15	EXO70I1-V	1455	484	126–480	5.61	42.48

Abbreviations: ORF, open reading frame; AA, amino acids; DL, PFam03081 domain location; PI, protein isoelectric point; MW, protein molecular weight.

**Table 2:** Number of *EXO70* from different species in each of the chromosomes.

Chromosome	*T. aestivum*			*T. dicoccoides*		*Ae. tauschii*	*H. vulgare*	Total
	A	B	D	A	B	D	H	
Chr.1	1	1	3	1	1	1	1	9
Chr.2	5	8	4	8	6	6	7	44
Chr.3	4	5	3	4	3	4	4	27
Chr.4	1	3	2	2	2	2	2	14
Chr.5	3	3	3	2	2	3	3	19
Chr.6	1	2	0	1	1	1	1	7
Chr.7	7	7	7	7	7	9	7	51
Total	22	29	22	25	22	26	26	171
*Unknow	2						1	3

* The genes that were assigned to unknown chromosome.

**Table 3:** Numbers of *EXO70* paralogs encoded by the surveyed genomes in total and individual subgroups.

Genome	Total Number	Subgroup								
		A	B	C	D	E	F	G	H	I
*H. villosa* (VV)	15	3	1	1	2	1	3	2	1	1
*H. vulgare* (HH)	26	4	2	2	2	2	5	3	1	5
*T. urartu* (AA)	26	3	2	2	2	1	5	1	1	9
*Ae. tauschii* (DD)	26	4	2	2	2	1	4	3	1	7
*T. dicoccoides* (AABB)	22–25 (47)	3 (6)	2 (4)	2 (4)	2 (4)	1 (2)	4 (8)	3 (6)	1 (2)	7 (11)
*T. aestivum* (AABBDD)	22–29 (75)	4 (12)	2 (5)	2 (6)	2 (5)	1 (3)	5 (13)	3 (6)	1 (3)	14 (22)
*B. distachyon* (Bd)	22	4	2	2	2	1	4	3	1	3
*Oryza sativa*	41	4	3	2	2	1	5	3	5	16
*Arabidopsis thaliana*	23	3	2	2	3	2	1	2	8	0
Total	238 (301)	32 (43)	18 (23)	17 (23)	19 (24)	11 (14)	36 (48)	23 (29)	20 (23)	64 (74)

Numbers in brackets indicate number of copies for polyploid genomes.

## Supplementary Materials

Supplementary materials can be found at http://www.mdpi.com/1422-0067/20/1/60/s1.

## Abbreviations

ABA	abscisic acid
DNA	deoxyribonucleic acid
EXPO	exocyst-positive organelle
ET	ethephon
GFP	green fluorescent protein
H_2_O_2_	hydrogen peroxide
MeJA	methyl jasmonate
PM	plasma membrane
Pm	powdery mildew
qRT-PCR	quantitative real time polymerase chain reaction
RNA	ribonucleic acid
SNARE	soluble NSF attachment protein receptor
SA	salicylic acid
